# Combined cord blood metabolomic and clinical signatures discriminate small for gestational age neonates in preeclampsia

**DOI:** 10.3389/fendo.2026.1873175

**Published:** 2026-08-31

**Authors:** Luhan Zhang, Weibo Huang, Shuyuan Xue, Xiaolan Chi, Gang Guo, Guifeng Ding

**Affiliations:** 1 Xinjiang Clinical Research Center for Perinatal Diseases, Urumqi Maternal and Child Health Hospital, Urumqi, China; 2 School of Medicine, Shihezi University, Shihezi, Xinjiang, China

**Keywords:** 2-Oxoadipic acid, caffeine metabolism, enrichment analysis, metabolomics, preeclampsia, small for gestational age infants

## Abstract

**Background:**

Preeclampsia (PE) subjects the fetus to a hostile intrauterine environment and elevates the risk of small for gestational age (SGA). The link between cord blood metabolic signs and PE exposure is still unclear. This connection may affect fetal growth.

**Methods:**

We did untargeted metabolomics profiling of cord blood. This involved 37 pregnancies with preeclampsia (PE) and 48 control pregnancies. We used liquid chromatography-mass spectrometry for the analysis. Researchers examined metabolites that showed differential expression. They also analyzed pathway enrichment. We used Spearman’s correlation to assess associations between metabolites and clinical indicators, including birth weight. We created a discriminative model for SGA. It combines metabolic features and clinical variables.

**Results:**

In the PE group, 205 metabolites changed significantly. Of these, 82 were upregulated and 123 were downregulated. Caffeine metabolism, lysine biosynthesis, and sulfur metabolism were the top three pathways affected. Among the dysregulated metabolites, 2-oxoadipic acid is important in lysine breakdown. It showed a strong negative link with birth weight (*r* = -0.396, *P* < 0.001). Xanthine had positive links to gestational age at delivery (*r* = 0.292, *P* = 0.007) and birth weight (*r* = 0.219, *P* = 0.044). Lysine was positively correlated with maternal BMI at delivery (*r* = 0.414, *P* < 0.001). Caffeine metabolites, like paraxanthine, theobromine, and theophylline, were enriched together. However, none showed a significant link to birth weight. The SGA discriminative model reached an AUC of 0.896 (*P* < 0.01).

**Conclusions:**

PE greatly changes the cord blood metabolome. This has a primary impact on caffeine, lysine, and sulfur metabolism. 2-Oxoadipic acid is linked to fetal growth restriction. Combining cord blood metabolic signatures with clinical data makes a strong model. These findings reveal metabolite-level disturbances that may help elucidate the metabolic basis of impaired fetal growth in preeclampsia.

## 1 Introduction

Preeclampsia (PE) is a multisystem disorder that occurs after 20 weeks of gestation, characterized mainly by hypertension, proteinuria, and multi-organ dysfunction (1). Its incidence rate is approximately 3% to 8%, and it is one of the main causes of perinatal mortality worldwide (2). Despite decades of research on PE, the primary mechanisms underlying this disorder remain elusive, with its onset likely resulting from the interplay of multiple factors. Notably, the symptoms of PE are typically alleviated following delivery and placental detachment, supporting the widely held view that placental abnormalities are central to PE pathogenesis (3). Among these, inadequate remodeling of the placental uterine spiral arteries is a relatively well-established contributor (4). These arteries serve as the conduit for maternal blood and nutrient supply to the fetus and placenta. Insufficient spiral artery remodeling leads to vessel narrowing and increased resistance, resulting in impaired placental perfusion (5) and subsequent functional deficits. Placental ischemia is thus a pivotal factor in PE development, and as pregnancy progresses, inadequate perfusion becomes more pronounced. Chronic ischemia may induce secondary atherosclerotic-like vascular changes (6), and abnormal hemodynamics may promote microthrombus formation and placental infarction (7). In later pregnancy, the ischemic placenta may release inflammatory mediators in response to hypoxia, adversely affecting maternal endothelial function. The hallmark clinical features of hypertension and proteinuria reflect underlying vascular endothelial dysfunction and disturbed regulation of vascular tone and permeability (8). Importantly, PE is not solely a vascular disorder but also involves significant endocrine and metabolic dysregulation during pregnancy. Placental ischemia-induced inflammation and oxidative stress may exacerbate maternal metabolic disturbances, such as insulin resistance and lipid metabolism abnormalities. These metabolic changes interact synergistically with endothelial dysfunction, collectively driving disease progression (9).

PE as a severe pregnancy-related complication, is far more than just a simple maternal disease. A large body of evidence indicates that the pathological changes, such as placental dysfunction, systemic inflammation (10), and oxidative stress (11) caused by PE will directly affect the intrauterine environment of the fetus through the placental barrier, thereby leading to a series of short-term and long-term adverse health outcomes. These effects include, but are not limited to: fetal growth restriction (FGR) (12), premature birth (13), low birth weight, and an increased risk of long-term cardiovascular metabolic diseases (14) and neurodevelopmental disorders (15) after birth. Epidemiologic data indicate that only approximately one-third of infants born to preeclamptic mothers are growth-restricted, suggesting that additional or distinct mechanisms operate at the maternal-fetal interface to determine whether small for gestational age(SGA) develops in the context of PE (16). However, there is currently no systematic understanding of how PE alters fetal growth trajectories through specific metabolic pathways, particularly regarding which metabolites play a pivotal role in this process.

Umbilical cord blood directly reflects the fetal circulatory and metabolic status at the time of delivery, making it an ideal biological sample for studying the effects of preeclampsia on fetal metabolic programming. Metabolomics Using high-throughput technology, it quantitatively measures the metabolic changes caused by pathological and physiological alterations, analyzing the changes of all endogenous small-molecule substances in the body at a certain moment. It is a research method for determining the overall state of an organism. Its research object is the collection of molecular compounds involved in the body’s metabolism. The main research target is small molecule metabolites (with a relative molecular weight of ≤ 1000). The research samples include blood, urine, different biological fluids, tissue samples, etc. The number of detections and detection difficulty are significantly lower than those of genes, making it more cost-effective (17).

In recent years, a small number of studies have utilized this technology to analyze the metabolic profiles of maternal plasma and umbilical cord blood in PE: The FINNPEC cohort identified significant abnormalities in acylcarnitines, trimethyl compounds, and urea cycle metabolites in PE umbilical cord blood, suggesting that fetal mitochondrial fatty acid β-oxidation and energy metabolism may be disrupted (18); Metabolomic studies of umbilical cord blood conducted at Peking Union Medical College Hospital further revealed disruptions in the sphingolipid and glycine/serine/threonine metabolic pathways (19); recent lipidomic studies have also identified metabolic remodeling of choline and glycerophospholipids in PE umbilical cord blood, which is associated with neonatal birth weight percentiles and length percentiles (20). Although the association between PE and SGA has been well documented in the literature, existing studies on umbilical cord blood metabolomics have primarily focused on PE as a maternal exposure condition. The metabolomic characteristics of PE-associated SGA, as an independent research phenotype, have not yet been fully characterized.

Based on this, this study aims to employ non-targeted metabolomics techniques to analyze umbilical cord blood samples from newborns of mothers with PE and those from normal pregnancies. The goal is to generate a comprehensive metabolic profile of fetal umbilical cord blood following PE exposure, identify differentially expressed metabolites, and pinpoint key metabolic pathways through KEGG pathway enrichment analysis. Furthermore, Spearman’s correlation analysis will be used to quantify the strength of the association between core differentially expressed metabolites and birth weight. The aim is to elucidate the mechanisms of intrauterine programming underlying PE-induced fetal growth restriction at the metabolic level and to provide a scientific basis for the subsequent identification of early metabolic biomarkers for discriminating PE-associated SGA infants.

## 2 Materials and methods

### 2.1 Study design and population

This research included singleton pregnancies delivered at Urumqi Maternal and Child Health Hospital in China from January to August 2024. Cord serum samples were collected from women with PE (n = 37) and from those with normal pregnancies (n = 48). PE was diagnosed according to Guidelines for the Diagnosis and Treatment of Hypertensive Disorders of Pregnancy (2020). Women in the PE group had the following characteristics after 20 weeks of gestation: ① Systolic blood pressure (SBP) ≥ 140 mmHg and/or diastolic blood pressure (DBP) ≥ 90 mmHg on two occasions at least 4 h apart; ② proteinuria ≥ 300 mg/24 h or severe features (thrombocytopenia, impaired liver function, persistent epigastric pain, renal insufficiency, pulmonary edema, new-onset headache, visual disturbances).The control group consisted of women with uncomplicated, normotensive pregnancies who delivered at term. Exclusion criteria for both groups included: multiple gestation, pre-existing chronic hypertension, pre-gestational diabetes mellitus, known fetal chromosomal or structural anomalies, and clinical chorioamnionitis. Because systematic antenatal ultrasound for fetal growth was not part of the protocol, non-hypertensive fetal growth restriction could not be assessed at enrollment and was therefore not applied as an exclusion criterion. SGA status was assigned postnatally according to birth weight percentile. Blood pressure values used in the discriminant model were recorded on admission for delivery. Gestational age was determined based on an early prenatal ultrasound examination. SGA was defined as birth weight below the 10th percentile for gestational age and sex, based on the Chinese national standard WS/T 800-2022 (Growth standards for newborns at different gestational ages at birth). It was approved by the Ethics Committee of Urumqi Maternal and Child Health Hospital (approval number: XJFYLL2023075). All women provided written informed consent.

### 2.2 Specimen collection

Umbilical cord blood specimens were collected from the venous side immediately following delivery. Blood samples were transferred into serum separator tubes and allowed to clot at ambient temperature for 30–60 minutes. Subsequently, the samples were centrifuged at 3,000 rpm for 10 minutes at 4 °C. The resulting supernatant serum was collected, aliquoted into 200 μL portions, and immediately stored at −80 °C until analysis.

### 2.3 Metabolite extraction

A 100 μL aliquot of each serum sample was mixed with 400 μL of a chilled extraction solvent (methanol: acetonitrile = 1:1, v/v) containing a stable isotope-labeled internal standard mixture. The mixture was transferred to a 96-well protein precipitation plate, shaken at 750 rpm for 5 min, and then allowed to rest for 5 min. The filtrate was collected into a collection plate. A pooled quality control (QC) sample was prepared by mixing equal volumes of supernatant from all study samples and was injected periodically throughout the analytical batch for data quality assessment and signal correction.

### 2.4 Untargeted LC-MS analysis

Metabolic profiling was performed on an Ultra-High Performance Liquid Chromatography (UHPLC) system, Vanquish, Thermo Fisher Scientific, coupled to a high-resolution mass spectrometer, Q-Exactive HF-X Hybrid Quadrupole-Orbitrap, Thermo Fisher Scientific. Chromatographic separation was achieved using a Waters ACQUITY UPLC BEH Amide (2.1 mm x 50 mm, 1.7 μm). The chromatographic column is used to separate the target compounds. In liquid chromatography, the mobile phase A consists of an aqueous solution containing 25 mmol/L ammonium acetate and 25 mmol/L ammonia solution, while the mobile phase B is acetonitrile. Sample tray temperature: 4 °C; injection volume: 2 μL.

The Orbitrap Exploris 120 mass spectrometer is capable of acquiring first- and second-stage mass spectrometry data under the control of the Xcalibur software (version 4.4, Thermo). The detailed parameters are as follows: Sheath gas flow rate: 50 Arb, Aux gas flow rate: 15 Arb, Capillary temperature: 320 °C, Full ms resolution: 60000, MS/MS resolution: 15000, Collision energy: SNCE20/30/40, Spray Voltage: 3.8 kV (positive)-3.4 kV (negative).

### 2.5 Data processing and metabolite identification

Features with a relative standard deviation greater than 30% in the QC samples or absent in more than 80% of the samples were removed. Metabolites were identified by matching accurate mass, retention time (RT), and MS/MS fragmentation patterns against in-house databases and public spectral libraries. Metabolite identification complied with the Metabolomics Standards Initiative (MSI) criteria, with all annotated metabolites assigned to four confidence tiers:

Level 1 (Confirmed identity, n=482): Matching RT, MS1 and MS2 spectra against authentic chemical standards under consistent instrumental conditions;
Level 2 (Putative identity, n=1173): MS1/MS2 spectral matching to HMDB and METLIN without standard RT validation;
Level 3 (Tentative candidates, n=73): Chemical class classification based on theoretical mass and predicted RT from public databases;
Level 4 (Unknowns, n=30825): Ion features that could not be matched to any recorded metabolites in the current database.

### 2.6 Statistical analysis

Data processing and univariate analyses were performed using SPSS 27.0 and R version 4.4.2. Normally distributed data were expressed as mean ± standard deviation and compared using the independent-samples t-test. Non-normally distributed data were expressed as median with interquartile range (*P_25_, P_75_*) and compared using the Mann–Whitney U test. Categorical data were presented as frequencies (n, %) and compared using the chi-squared (*χ²*) test. For untargeted metabolomic data, multiple testing correction was performed via the Benjamini–Hochberg false discovery rate (FDR) procedure, with a predefined threshold of FDR < 0.05 to identify significantly differential metabolites.

Multivariate statistical analysis was employed to visualize metabolic profile disparities. Unsupervised Principal Component Analysis (PCA) was performed to overview intrinsic clustering and identify potential outliers. Supervised Orthogonal Projections to Latent Structures-Discriminant Analysis (OPLS-DA) was implemented to maximize group separation. The model’s goodness of fit (R²Y) and predictive ability (Q²) were assessed using a default 7 round cross validation, and its robustness was validated by a 200-permutation test. Discriminative metabolites were selected based on a combined criterion of VIP > 1.0 from the OPLS-DA model and univariate FDR < 0.05.

To further explore the association between metabolic features and the occurrence of small for gestational age infants within the preeclamptic group, an explanatory discriminant model was constructed. The candidate predictors included maternal age, gestational age at delivery, blood pressure, body mass index, and the top discriminative metabolites. Model performance was assessed by the area under the receiver operating characteristic curve (AUC). A two-tailed *P* value < 0.05 was considered nominally significant where applicable.

## 3 Result

### 3.1 Comparison of baseline characteristics and pregnancy outcomes

The baseline characteristics and pregnancy outcomes in different groups of pregnant women are shown in **Table 1**. Between the PE and Control groups, there were no significant differences in gravidity, parity, firstborn rates, and neonatal Apgar score/1 minute. There were significant differences in gestational age at delivery, premature birth rate, and SGA between the two groups. The SBP, DBP, and BMI of the PE group were significantly higher than those of the Control group. The birth weight of the PE neonates was significantly lower than that of the Control neonates.

**Table 1:** Comparison of baseline information and pregnancy outcomes in different group of pregnant women.

Parameters	PE group(37)	Control group(48)	*t*/*t^’^*/*χ^2^*/Z	*P*
Age at delivery,year	34.05 ± 0.75	30.58 ± 0.60	-3.68	<0.01
Gravidity(P_25_,P_75_)	2(1,3)	2(1,3)	-0.06	0.95
Parity(P_25_,P_75_)	2(1,2)	2(1,2)	-0.75	0.46
Firstborn,n(%)	2(5.4)	1(2.1)	0.68	0.41
BMI at Delivery(kg/m²)	32.44 ± 5.07	27.25 ± 3.32	-5.39	<0.01
GA at birth, weeks(P_25_,P_75_)	37(36,38)	39(38,40)	-5.89	<0.01
Birth weight, g	3025.14 ± 873.78	3439.17 ± 410.51	0.27	0.01
<37 weeks	2313.57 ± 792.39	3190.00 ± 0.00	-1.07	0.31
≥37 weeks	3458.26 ± 599.99	3444.47 ± 413.28	0.11	0.91
premature birth rate, n(%)	14(37.8)	1(2.1)	18.38	<0.01
SGA, n(%)	7(18.9)	2(4.2)	4.80	0.03
Apgar/1 min(P_25_,P_75_)	10(9,10)	10(10,10)	-1.52	0.13
SBP, mmHg(P_25_,P_75_)	132(121,141)	120(112,126)	-3.59	<0.01
DBP, mmHg(P_25_,P_75_)	82(74,93)	77(70,80)	-2.78	<0.01
GDM, n(%)	9(24.3)	15(31.3)	0.50	0.48

BMI, Body mass index; GA, Gestational age; SGA, Small for gestational age; SBP, Systolic blood pressure; DBP, Diastolic blood pressure; GDM, Gestational diabetes mellitus.

### 3.2 Quality control and multivariate statistical analysis

**Figure 1:**
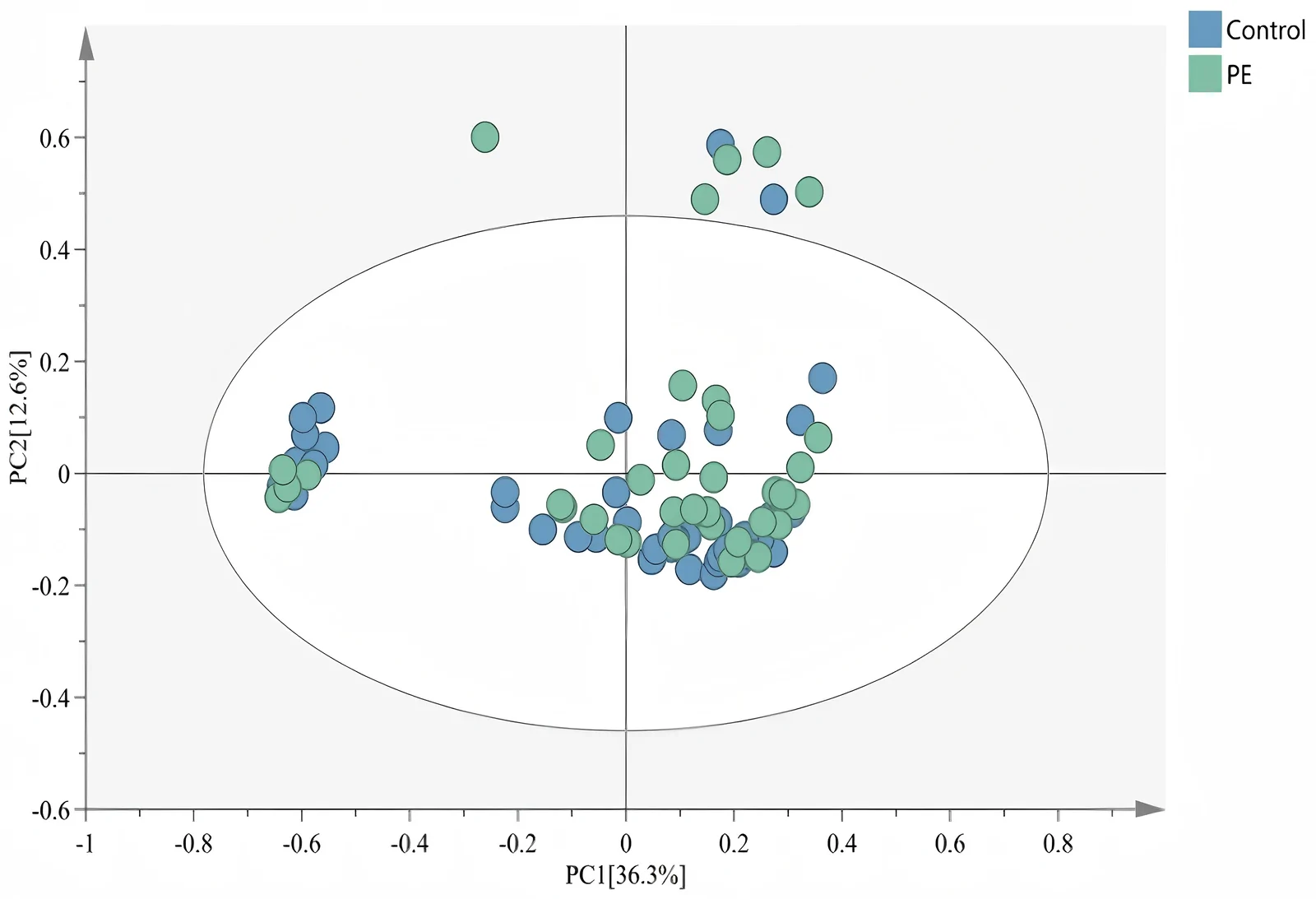
Principal component analysis (PCA) plots show some separation of metabolic profiles between control (in blue) and PE (in green). PCA model: R^2^X = 0.560, Q^2^ = 0.513.

**Figure 2:**
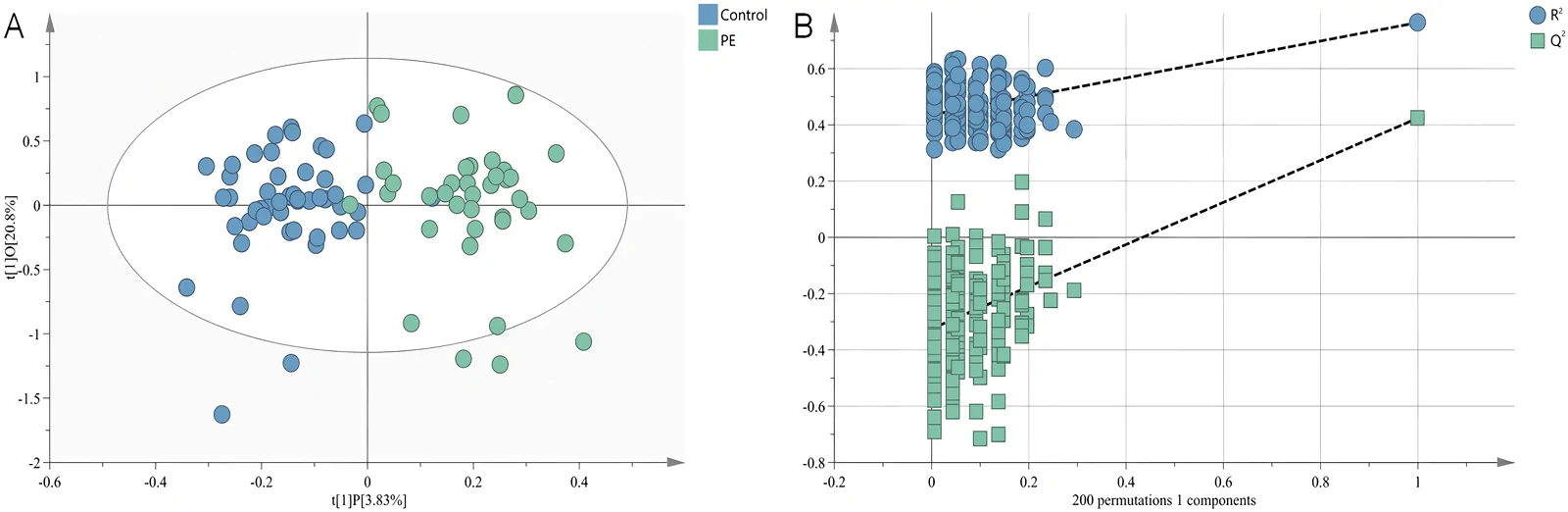
Orthogonal partial least squares discriminant analysis (OPLS-DA) plots show significant separation of metabolic profiles between control (in blue)and PE (in green). **(A)** OPLS-DA model, R^2^X = 0.246, R^2^Y = 0.764, Q^2^ = 0.424. **(B)** A permutation test of OPLS-DA model. The Y-axis intercepts were: R^2^Y (0, 0.436), Q^2^(0,–0.325).

After data processing, 1340 metabolites were detected on the NGM platforms. PCA of data from the platform showed that the PE and Control group neonatal metabolomic profiles were separated to some extent (**Figure 1**). OPLS-DA analysis on the NGM platform showed that we can distinguish the PE and Control group according to the metabolomics data (**Figure 2A**). Permutation on the NGM platform showed no overfitting in the OPLS-DA model (**Figure 2B**).

### 3.3 Screening and identification of differential metabolites

**Figure 3:**
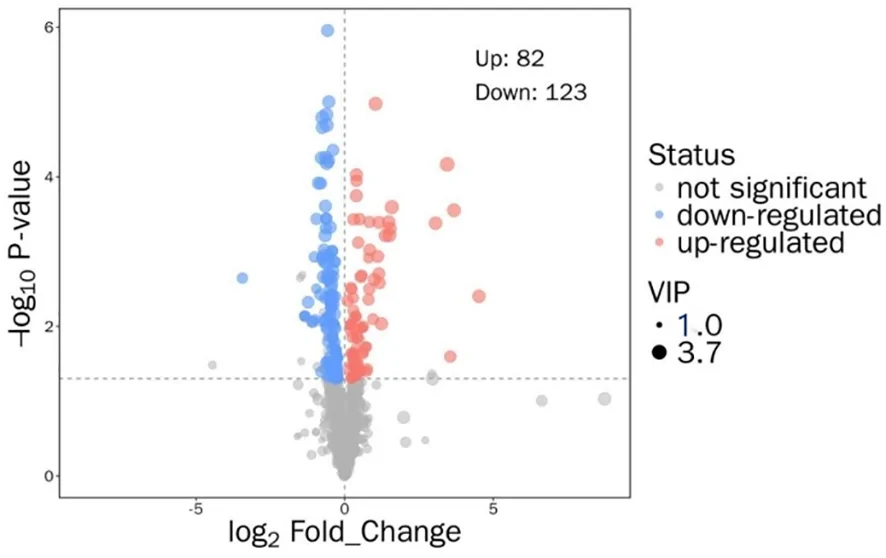
Volcano plot of differentially expressed metabolites between the PE group and the normal pregnancy group. Each point in the volcano plot represents a metabolite. The x-axis represents log_2_FC, the y-axis represents the P-value from the t-test (as the negative of the logarithm to base 10), and the size of the scatter plot represents the VIP value from the OPLS-DA model; larger scatter plots indicate higher VIP values. Significantly upregulated metabolites are shown in red, significantly downregulated metabolites are shown in blue, and metabolites with no significant difference are shown in gray.

**Figure 4:**
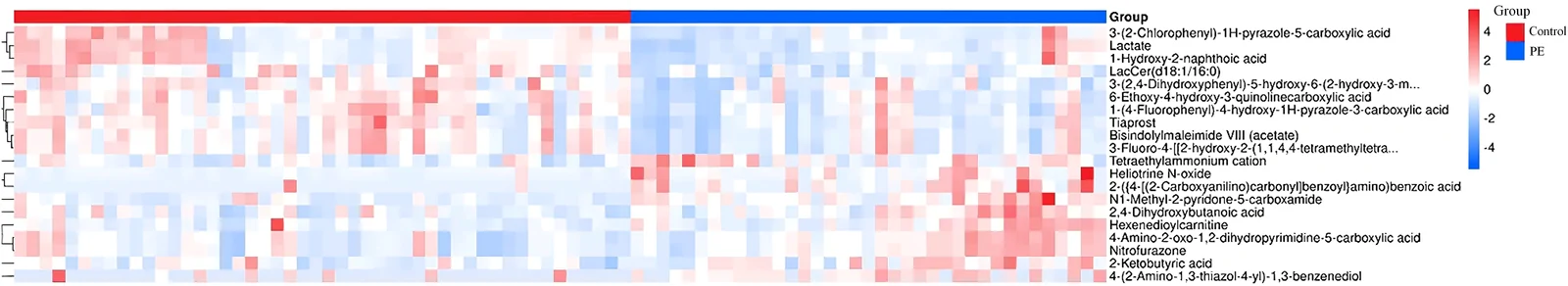
The heatmap displays the top 10 up-regulated and top 10 down-regulated differential metabolites. The color blue indicates decreasing expression, and red indicates increasing expression. The color intensity reflects the corresponding abundance difference.

**Figure 5:**
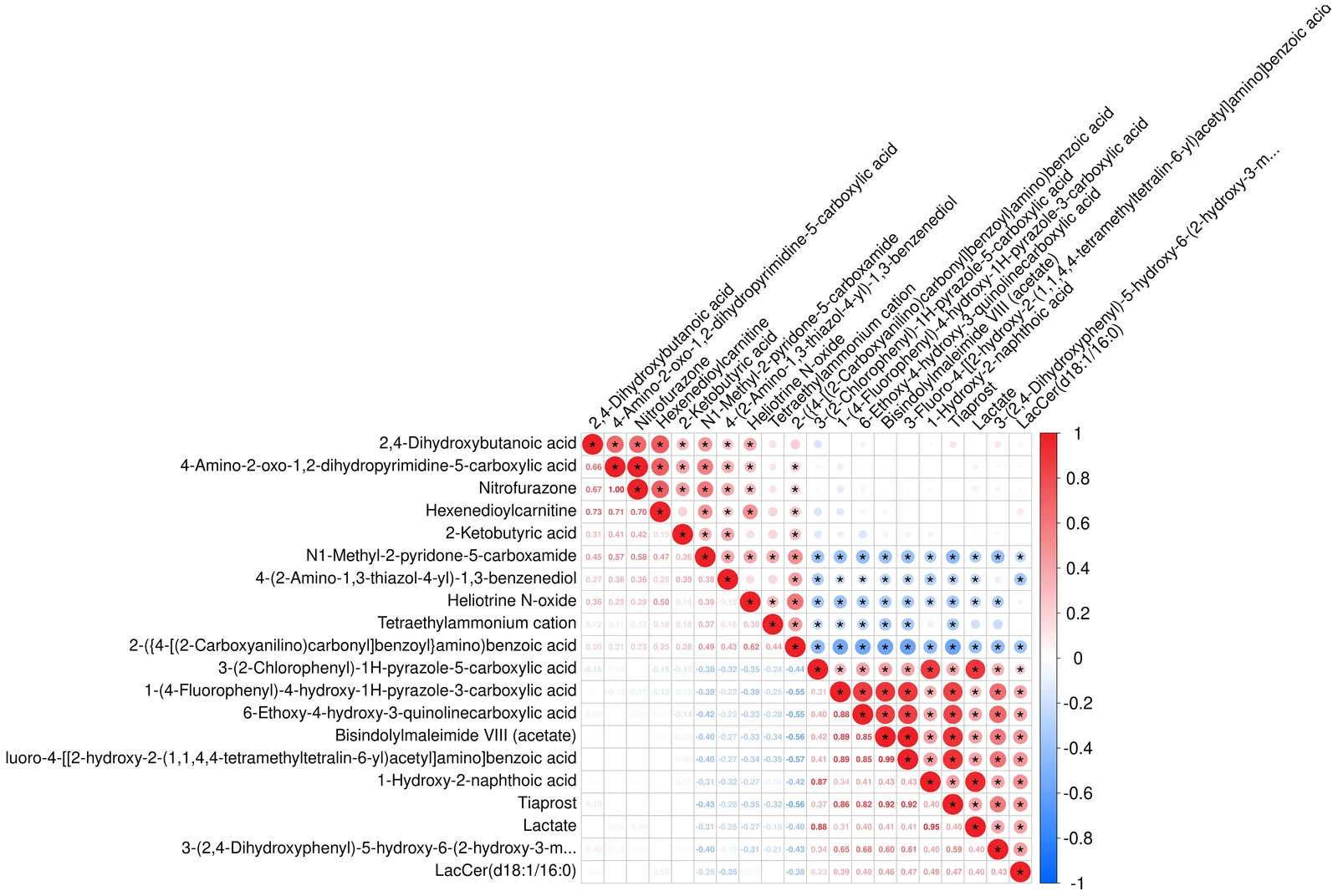
Heatmap of correlation analysis between the control group and the PE group. The x- and y-axes in the figure represent the metabolites compared within each group. The colored blocks at different positions indicate the magnitude of the correlation coefficients between the corresponding metabolites; red indicates a positive correlation, blue indicates a negative correlation, and darker shades indicate a stronger correlation. Significant correlations are marked with an asterisk (*).

By combining the results of multivariate and univariate statistical analyses (VIP > 1 and P < 0.05), we identified 205 metabolites with significant differences. Full details of these differential metabolites are provided in **Supplementary Table S1**. We visualized the results of the differential metabolite screening using a volcano plot, as shown in **Figure 3**. Hierarchical cluster analysis was performed on the screened differential metabolites. The heatmap (**Figure 4**) showed that the top 20 differential metabolites could be clustered into two types, enriched in umbilical cord serum of the PE and Control groups. In the NGM platform, 82 metabolites increased in the PE group (including 6 lipid-related metabolites, 12 amino acid-related metabolites, and others), and 123 decreased in PE group(including 6 lipid-related metabolites, 9 amino acid-related metabolites, and others).We calculated the correlation coefficients for the quantitative values of differentially expressed metabolites using Spearman’s correlation method and presented the results as a heatmap. Taking the comparison between the normal group and the PE group as an example, we selected the top 10 significantly upregulated and downregulated metabolites, respectively; the results are shown in **Figure 5**. The underlying raw matrix for heatmap visualization is provided in **Supplementary Table S2**.

### 3.4 KEGG metabolic pathway enrichment analysis of preeclampsia

**Figure 6:**
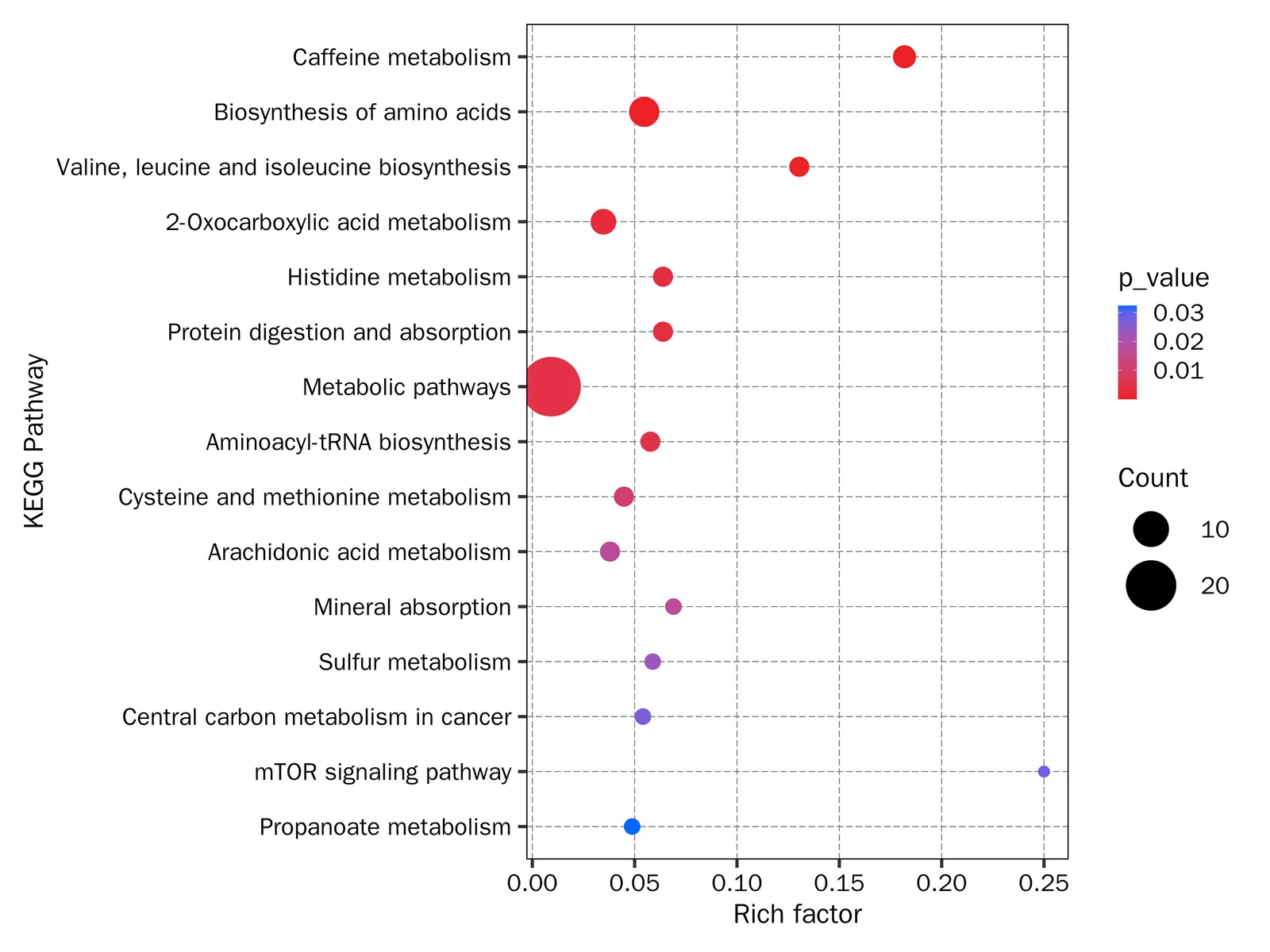
KEGG enrichment plot of differentially expressed metabolites between the PE group and the normal pregnancy group. The x-axis represents the Rich Factor for each pathway, and the y-axis shows the names of the KEGG metabolic pathways. The size of the dots indicates the number of differentially expressed metabolites enriched in that pathway. The color indicates the magnitude of the P-value; the smaller the P-value, the redder the color, indicating a more significant level of enrichment.

**Figure 7:**
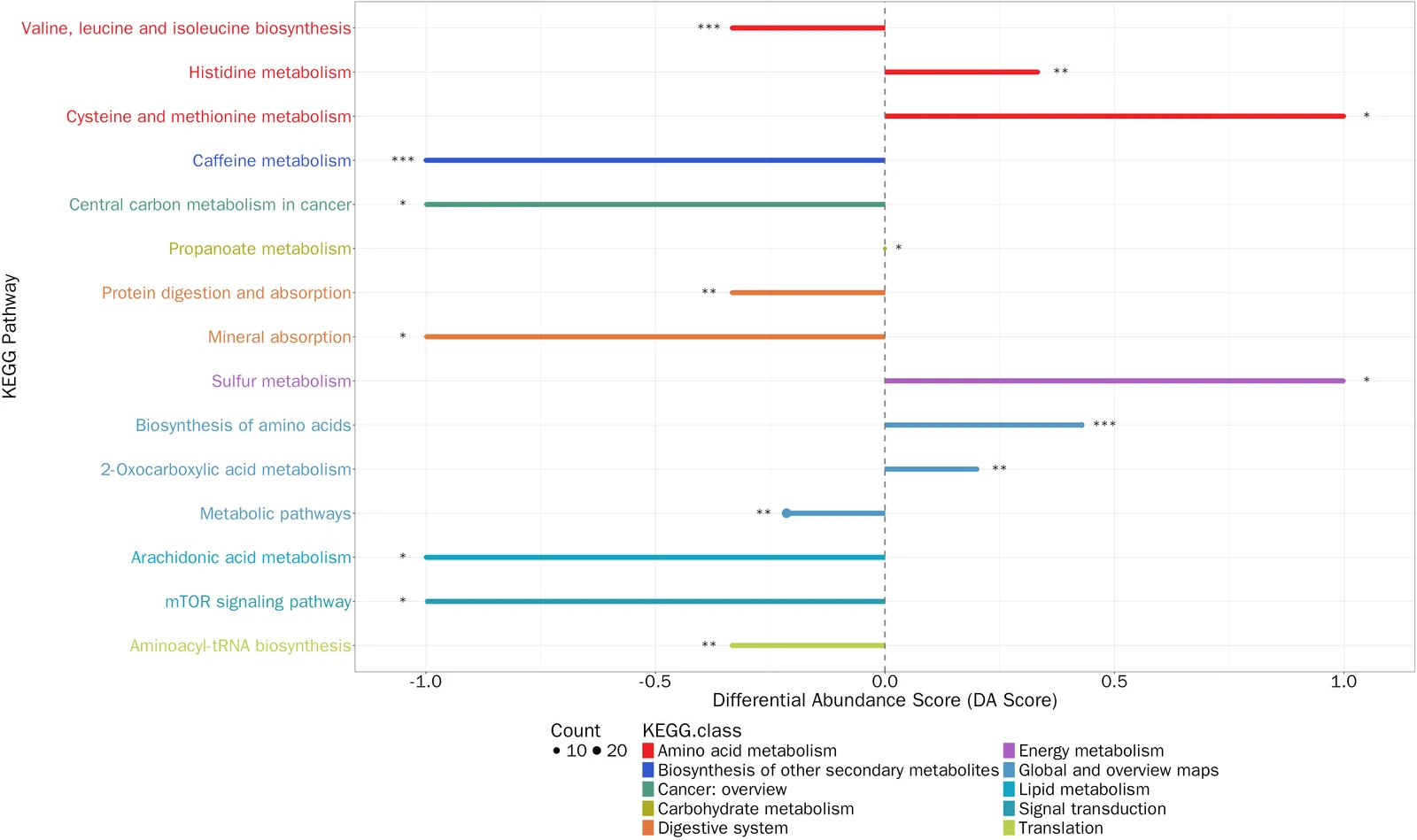
KEGG pathway enrichment analysis of significantly altered metabolites in umbilical cord serum between the preeclamptic and control groups. The y-axis lists the enriched pathways; the x-axis represents the pathway impact score. The color gradient corresponds to the −log_10_ *P*-value, with redder hues indicating higher statistical significance. Bubble/bar size reflects the number of differentially abundant metabolites mapped to each pathway.

**Figure 8:**
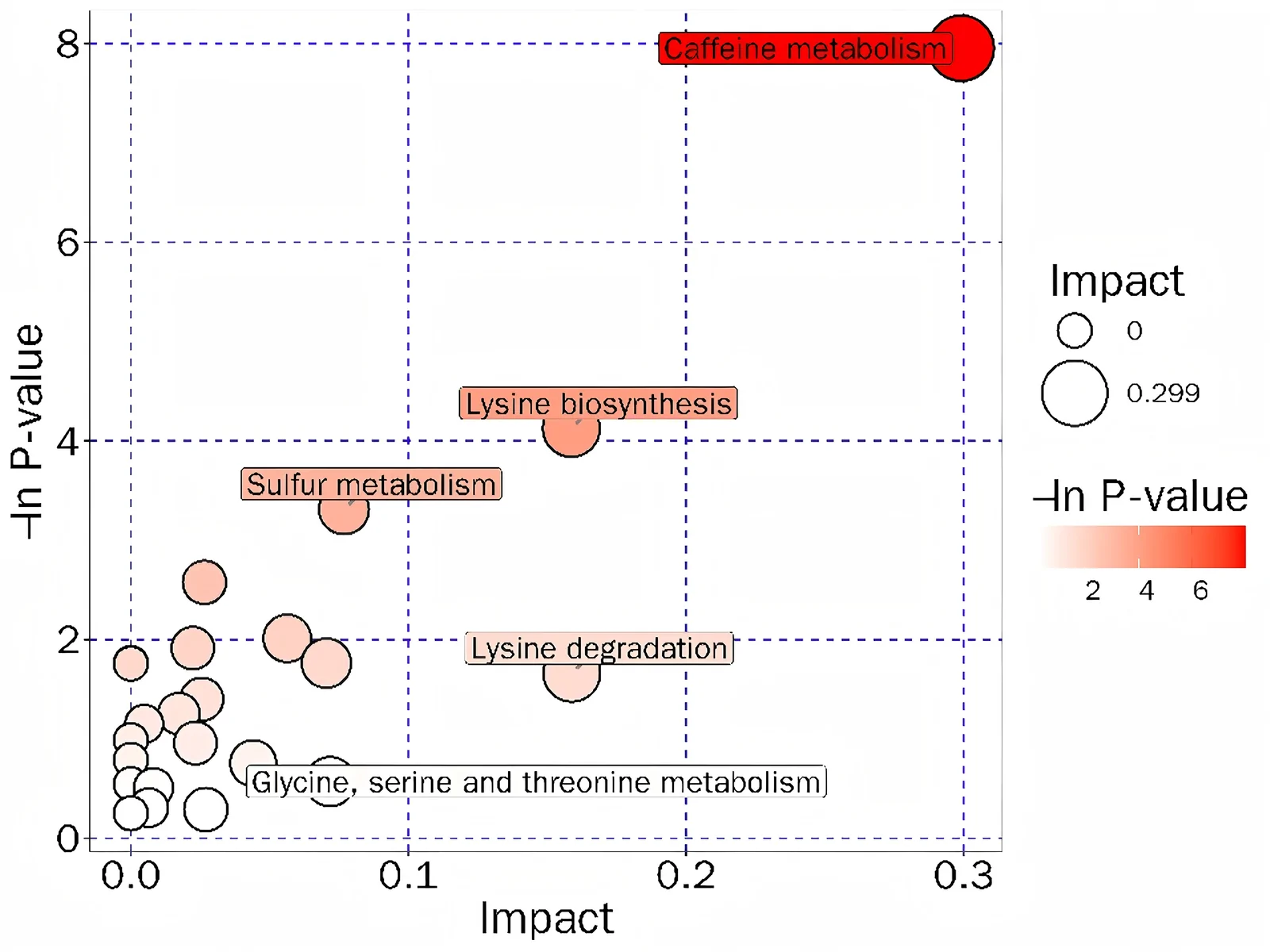
Metabolic pathway analysis results of PE group, presented as a bubble graph. Each bubble represents a metabolic pathway. The x-axis of the bubble and its size represent the impact factors of the pathway in the topology analysis. The y-axis of the bubble and its color represent the p-value of enrichment analysis [represented by the negative natural logarithm, -ln(*P*)]. The color intensity reflects the corresponding p-value and the enrichment degree.

To explore the biological functions of differentially expressed metabolites, 205 differentially expressed metabolites were integrated into pathway analysis. The raw outputs of this pathway‑enrichment analysis are provided in **Supplementary Table S3**. Based on the enrichment results of differentially expressed metabolites in KEGG metabolic pathways, the Rich Factor was calculated; a higher value indicates a greater degree of enrichment. The KEGG enrichment plot for differentially expressed metabolites is shown in **Figure 6**. Based on the KEGG metabolic database, we found that “Valine, leucine, and isoleucine biosynthesis” and “Caffeine metabolism” were the most significantly enriched pathways (Rich Factor > 0.1, P < 0.05), and we summarized the significantly differentially expressed metabolites within these pathways. The key differentially expressed metabolites mapped to these core pathways are listed in **Table 2**. The Differential Abundance Score is shown in **Figure 7**. By conducting a comprehensive analysis of the pathways associated with differentially expressed metabolites, we can further screen these pathways to identify the key pathways most strongly correlated with metabolic differences. First, we map the differentially expressed metabolites to authoritative metabolite databases such as KEGG and PubChem. After obtaining the matching information for these metabolites, we search the pathway databases for the corresponding species, Homo sapiens (human), and perform metabolic pathway analysis. The 5 pathways with the highest correlation are listed in **Table 3**. The topological impact factors and enrichment analysis p-values of the corresponding pathways are shown in the bubble graph (**Figure 8**). Using the screening criteria of P < 0.05 or pathway impact > 0.1, three core metabolic pathways closely associated with PE were identified: caffeine metabolism, lysine biosynthesis, and sulfur metabolism.

**Table 2:** Metabolites significantly enriched in different pathways.

KEGG ID	Differentially expressed metabolite name	Metabolic pathway	VIP	FC	Change	*P*
C00385	Xanthine	1	2.04	0.65	↓	<0.001
C00123	Leucine	2	1.36	0.85	↓	0.049
C13747	Paraxanthine	1	1.55	0.39	↓	0.007
C07480	Theobromine	1	1.55	0.39	↓	0.007
C07130	Theophylline	1	1.55	0.39	↓	0.007
C00047	Lysine	2	1.35	1.17	↑	0.032
C00322	2-Oxoadipic acid	2	2.01	1.07	↑	0.005

Pathway 1 is the caffeine metabolic pathway; Pathway 2 is the biosynthetic pathway for valine, leucine, and isoleucine.

**Table 3:** Metabolic pathways related to differential metabolites between PE and control group.

Pathways	Hits	*P*-value	-ln(*P*)	Impact
Caffeine metabolism	4	<0.01	7.95	0.30
Lysine biosynthesis	3	0.02	4.13	0.16
Sulfur metabolism	2	0.04	3.31	0.08
Lysine degradation	2	0.19	1.66	0.16
Glycine, serine and threonine metabolism	1	0.57	0.57	0.07

### 3.5 Determination of key metabolites

**Figure 9:**
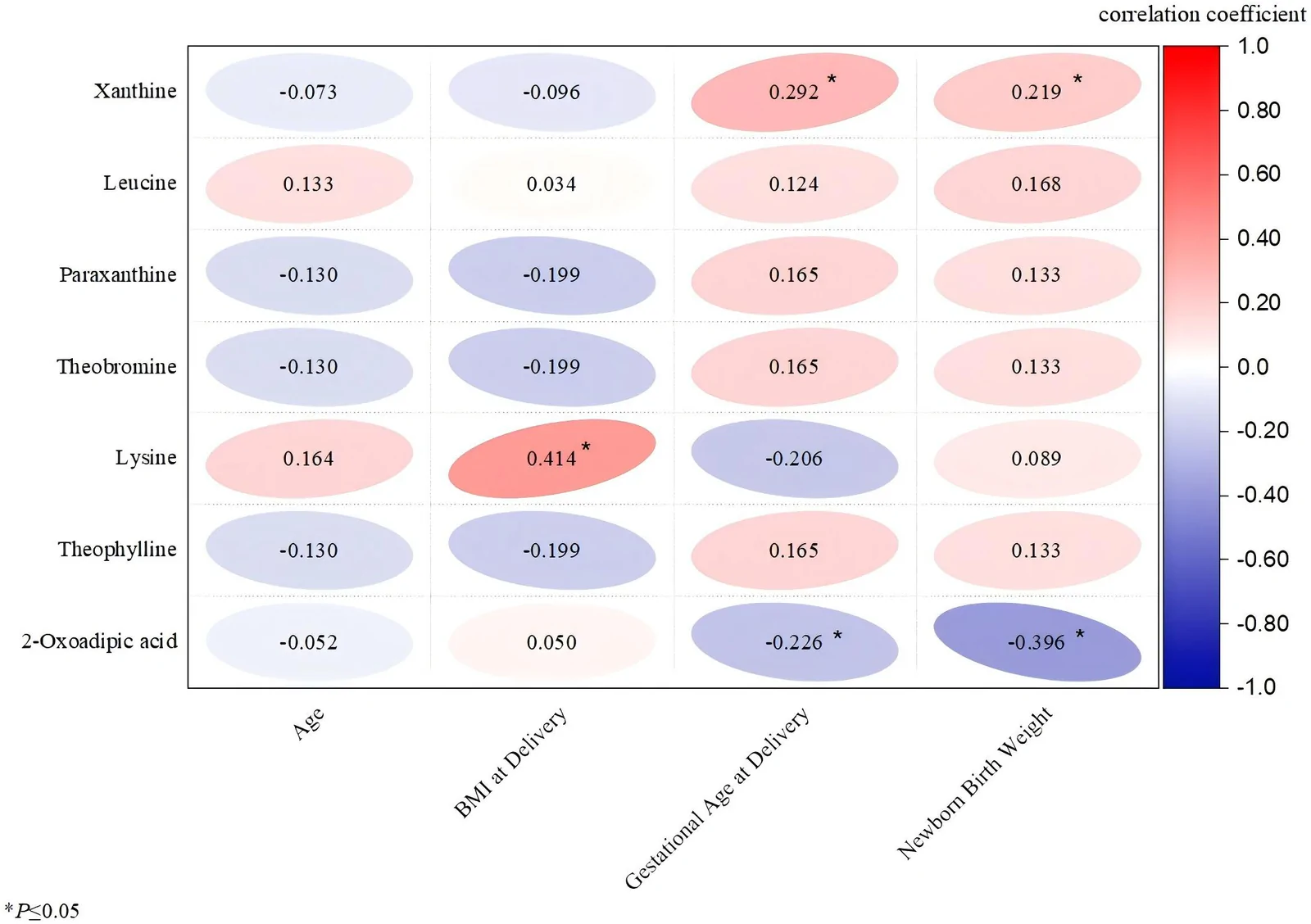
Heatmap of Pearson correlation coefficients between differential metabolites in umbilical cord serum and clinical variables. Red indicates positive correlations, blue indicates negative correlations, and correlation coefficients (r values) are labeled in each cell. Statistically significant correlations with *P* < 0.05 are marked with an asterisk (*).

To evaluate the association between metabolites significantly enriched in differential pathways and disease-related physiological characteristics, we performed Spearman’s correlation analysis between these metabolites and clinical indicators; the results are shown in **Figure 9**. Among baseline clinical indicators, xanthine showed a weak positive correlation with gestational age at delivery (r = 0.292, *P* = 0.007) and neonatal birth weight (r = 0.219, *P* = 0.044); Lysine showed a moderate positive correlation with BMI at delivery (r = 0.414, *P* < 0.001); 2-Oxoadipic acid showed weak to moderate negative correlations with gestational age at delivery (r = −0.226, *P* = 0.038) and neonatal birth weight (r = −0.396, *P* < 0.001).

After identifying the differentially abundant metabolites between the PE and control groups, five metabolites—homoserine, lysine, oxoadipic acid, xanthine, and paraxanthine—were initially selected based on their statistical significance (VIP > 1.0 and univariate *P* < 0.05) and their biological relevance to pathways implicated in placental function and fetal growth. Specifically, homoserine and lysine are central to amino acid metabolism and reflect alterations in protein turnover and nitrogen balance at the maternal–placental interface; oxoadipic acid, an intermediate in the degradation of lysine and tryptophan, suggests potential dysregulation of energy metabolism; and xanthine and paraxanthine, as purine metabolites and endogenous caffeine analogs, may indicate heightened oxidative stress associated with placental hypoxia/reperfusion. These five metabolites were therefore retained as candidate variables for multivariate analysis within the preeclamptic subgroup.

### 3.6 Construction of an explanatory discriminant model

**Figure 10:**
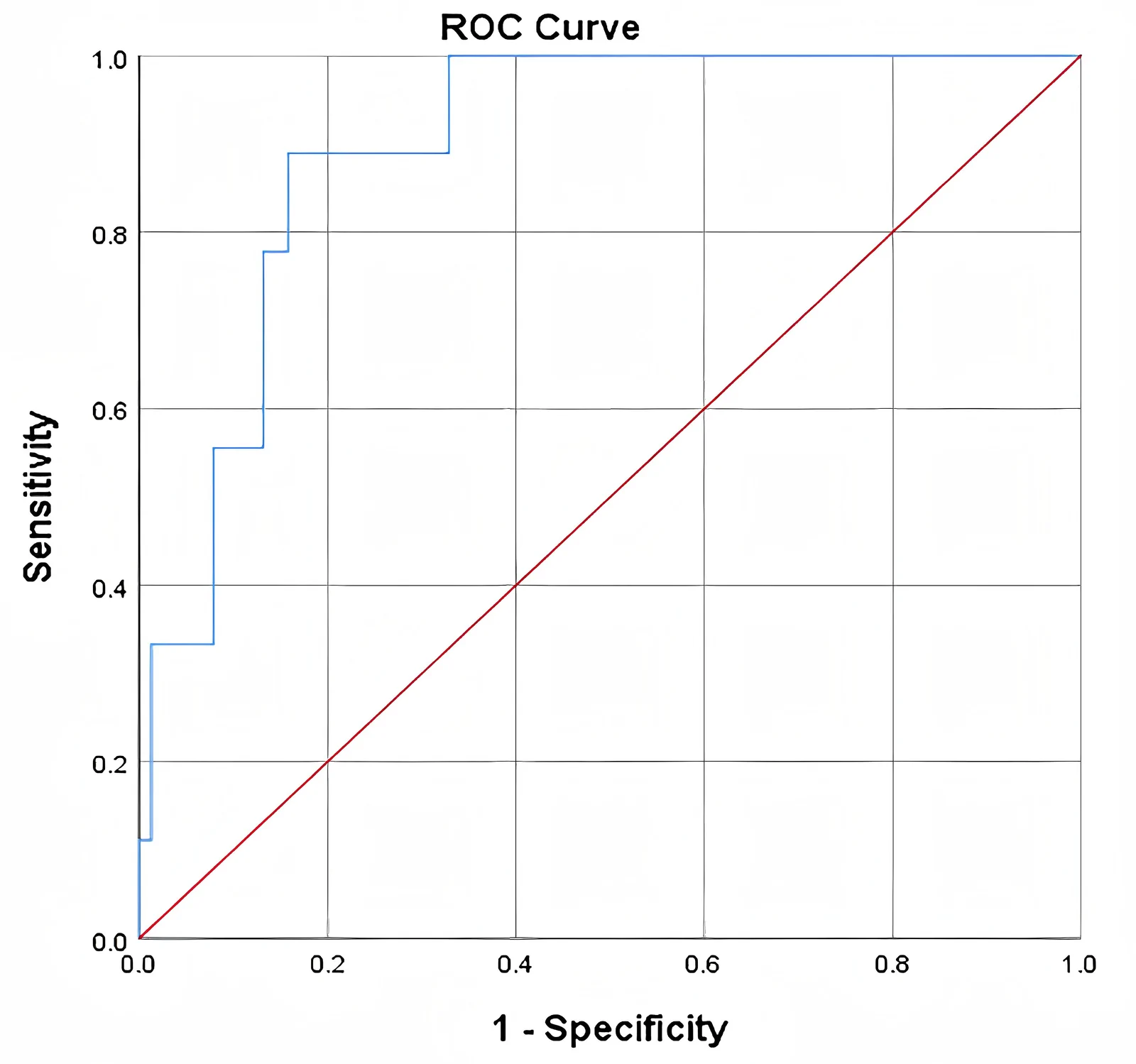
This receiver operating characteristic curve presents the model constructed based on indicators such as age, gestational age, systolic blood pressure, diastolic blood pressure, BMI, and 5 differential metabolites. [Area = 0.896, 95%CI(0.816,0.976), *P* < 0.01].

To examine the collective association between these metabolites-together with pertinent clinical variables and SGA status at delivery, an explanatory discriminant model was developed. Because umbilical cord blood metabolites and gestational age at delivery are only available after birth, the model was constructed not as a prenatal prediction tool, but rather to characterize the metabolic landscape that best discriminated SGA infants from appropriate-for-gestational-age (AGA) infants born to mothers with preeclampsia. Maternal age, gestational age at delivery, systolic blood pressure, diastolic blood pressure, body mass index, and the five candidate metabolites were entered into a stepwise logistic regression model. The discriminative performance of the model was assessed by the area under the receiver operating characteristic curve (AUC). The model achieved an AUC of 0.896 (95% CI: 0.816–0.976, *P* < 0.001), indicating good discriminatory ability in this cohort. The detailed results are presented in **Figure 10**. The full experimental metabolite intensity dataset is provided in **Supplementary Table S4**.

## 4 Discussion

In this study, we employed non-targeted metabolomics to identify metabolic reprogramming patterns associated with fetal growth restriction in umbilical cord blood from PE. We identified a total of 205 differentially expressed metabolites, and through pathway enrichment analysis, identified caffeine metabolism, lysine biosynthesis, and sulfur metabolism as the three pathways most significantly affected. Further analysis in conjunction with clinical phenotypes revealed that 2-oxohexanedioic acid was significantly negatively correlated with birth weight, while xanthine was positively correlated with gestational age and birth weight. More importantly, the SGA classification model constructed based on umbilical cord blood metabolic profiles and clinical indicators at the time of delivery achieved an AUC of 0.896 (*P* < 0.01), demonstrating excellent classification performance. These findings indicate that cord blood metabolomic signatures discriminate SGA from AGA infants in preeclamptic pregnancies through pathway-specific alterations in amino acid metabolism, purine handling, and energy substrate utilization, rather than merely reflecting differences in birth weight. The results provide new mechanistic insights into the metabolic perturbations associated with impaired fetal growth under PE exposure.

The results indicated that it was mainly the amino acids and lipids that underwent changes. About the amino acids, in the PE group, the concentrations of valine and leucine decreased, whereas the concentration of lysine increased. It’s similar to the study by Natasya Prameswari (21). However, the sample used in this study was maternal blood, which is different from the cord blood used in our current study. This indicates that maternal amino acid metabolism in preeclampsia is likely to affect the next generation through the umbilical cord. The differential lipid metabolites in this study did not show significant correlations, further research is needed.

Further, we use metabolites to conduct pathway analysis, and PE newborns were associated with the following pathways.

### 4.1 Caffeine metabolism

The caffeine metabolism in the human body is almost entirely dependent on the cytochrome P450 enzyme system in the liver: its core path is caffeine → paraxanthine → theobromine/theophylline → xanthine → uric acid. Paraxanthine, the primary metabolite of caffeine, is catalyzed by the CYP1A2 enzyme (accounting for approximately 95%) (22, 23). The down-regulation of its concentration is the most direct evidence indicating that the activity of the CYP1A2 enzyme is severely inhibited in patients with preeclampsia. Theobromine and theophylline are further metabolites of paraxanthine. Their reduction confirms that as the supply of upstream raw materials (parafxanthine) has decreased, the production of downstream products has naturally decreased as well. Xanthine is a further downstream product in the caffeine metabolic pathway and is also a common intermediate in endogenous purine metabolism (24). Caffeine metabolism downward adjustment has a dual significance. Firstly, exogenous metabolic obstruction: It indicates that the entire exogenous metabolic pathway from caffeine to xanthine is operating at a low speed. Moreover, endogenous metabolism may be affected: xanthine is also required for the catabolism of the body’s own purine nucleotides, such as ATP and GTP. Its down-regulation may imply that the energy metabolism and turnover of cells in PE patients may also be slowed down (decreased endogenous purine catabolism).Although correlation analysis showed that none of these three methylxanthines was individually significantly associated with birth weight, the overall disruption of the metabolic pathway suggests that fetal caffeine metabolism may be impaired in cases of preeclampsia. The placenta lacks an effective metabolic barrier against caffeine, and caffeine ingested by the mother can pass almost freely through the placenta into the fetal circulation (25). Epidemiological studies have indicated that high caffeine intake during pregnancy may increase the risk of FGR and low birth weight (26).In this study, the accumulation of caffeine metabolites in the umbilical cord blood of newborns with preeclampsia may reflect abnormal maternal-fetal exchange of substances caused by insufficient placental perfusion in the context of preeclampsia, leading to further retention of caffeine and its metabolites in the fetus; however, this hypothesis requires validation using paired maternal-umbilical cord blood samples and dietary survey data.

### 4.2 Lysine biosynthesis

Our findings indicate an aberrant activation of the lysine biosynthesis pathway in preeclampsia patients. This is likely a compensatory response to placental mitochondrial dysfunction and severe oxidative stress (27), which disrupts the normal flow of the pathway, causing a consequential accumulation of key intermediates like homoserine, lysine, and oxoadipic acid. This study found that 2-oxohexanedioic acid was significantly negatively correlated with birth weight (r = -0.396, *P* < 0.001), making it the most prominent metabolite associated with growth restriction to date. The accumulation of 2-oxohexanedioic acid may reflect an impairment in lysine catabolic pathways, a phenomenon that has been reported in diabetes and mitochondrial dysfunction (28). In preeclampsia, placental hypoxia and oxidative stress can lead to impaired mitochondrial function in fetal tissues, resulting in decreased activity of the 2-oxohexanedioate dehydrogenase complex and subsequent accumulation of its substrate, 2-oxohexanedioate. Furthermore, 2-oxohexanedioic acid has been shown to be an analog of 2-ketoglutarate, competitively inhibiting 2-ketoglutarate-dependent dioxygenases, and affecting DNA and histone demethylation, potentially linking metabolic dysfunction to fetal growth programming at the epigenetic level (29). At the same time, lysine itself showed a significant positive correlation with maternal BMI (r = 0.414, *P* < 0.001), suggesting that maternal nutritional status may indirectly influence fetal amino acid supply through umbilical cord blood metabolites, further supporting the association between the maternal metabolic environment and fetal metabolic reprogramming.

### 4.3 Sulfur metabolism

The significant upregulation of o-acetyl-l-serine and homoserine in the sulfur metabolism pathway observed in our preeclampsia study is primarily attributed to a compensatory response to oxidative stress. This phenomenon suggests that placental tissue may activate an antioxidant defense mechanism centered on glutathione synthesis to counteract excessive reactive oxygen species damage (30). The accumulation of o-acetyl-l-serine, as a direct biosynthetic precursor of cysteine, reflects the compensatory activation of the cysteine biosynthesis pathway to meet the metabolic demand for substantial glutathione production. The concurrent upregulation of homoserine, an upstream metabolite of o-acetylserine, further supports the global activation of this metabolic axis. The abnormal elevation of these two metabolites collectively reveals the crucial relationship between sulfur amino acid metabolic reprogramming and oxidative stress in the pathological state of preeclampsia. The enrichment of sulfur metabolism pathways in umbilical cord blood may reflect the fetus’s activation of compensatory sulfur metabolism pathways to counteract the oxidative burden originating from the maternal-placental interface. Although our results did not validate each sulfur metabolism metabolite individually, the significant enrichment of this pathway offers a potential direction for future targeted interventions.

Although the discriminant model developed in this study achieved an AUC of 0.896, we caution that it is not intended as a clinical tool for the reasons discussed above. Nevertheless, these findings raise the hypothesis that cord blood metabolic profiling might, in future studies, help refine the classification of SGA beyond birth weight alone. For instance, it is conceivable that among infants meeting the SGA criterion, distinct metabolic profiles could distinguish those with true pathological growth restriction from constitutionally small infants—a distinction that may have implications for postnatal follow-up. Similarly, it may be worth investigating whether a subset of AGA infants exposed to PE exhibit metabolic signatures resembling those of SGA infants, potentially indicating subclinical metabolic stress not reflected in birth weight. These hypotheses remain speculative and require validation in larger, longitudinally designed cohorts with detailed metabolic phenotyping and long-term follow-up data.

### 4.4 Limitation

Several limitations should be noted. First, the number of SGA infants was small (7 in the PE group and 2 in the controls), limiting statistical power and requiring external validation of the discriminant model in larger cohorts. Second, potential confounders—including maternal pre-pregnancy weight, diet, caffeine intake, smoking, alcohol use, socioeconomic status, and medications—were not systematically recorded and could not be adjusted for. Third, the absence of serial third-trimester fetal ultrasound data prevented differentiation of true fetal growth restriction from constitutional smallness. Fourth, untargeted metabolomics provides semi-quantitative data; key metabolites (e.g., oxoadipic acid) require absolute quantification by targeted methods for confirmation. Finally, with cord blood collected at delivery, precludes causal inference. The identified metabolite – SGA associations reflect concurrent metabolic features rather than prenatal predictors. Future studies employing longitudinally collected maternal biospecimens (e.g., mid-pregnancy plasma or urine) are needed to assess the potential of these pathways for early risk stratification.

## 5 Conclusion

In summary, we can observe that there are significant differences in amino acid metabolism between pregnant women with PE and the control group. Newborn umbilical cord blood exhibits metabolic reprogramming characterized by abnormalities in caffeine metabolism, lysine metabolism, and sulfur metabolism, with 2-oxohexanedioic acid serving as a key molecule linking lysine metabolism abnormalities to fetal growth restriction. The SGA classification model developed in this study by integrating umbilical cord blood metabolic profiles with clinical indicators demonstrated good classification performance, suggesting that umbilical cord blood metabolomics can serve as a complementary tool for the precise assessment of neonatal growth status. This finding supports the exploration of incorporating metabolomics into the paradigm of perinatal risk stratification. Future research should utilize multicenter prospective cohorts to validate the discriminatory performance of these metabolic biomarkers in independent validation sets. Concurrently, by integrating longitudinal follow-up data, the value of these biomarkers in predicting long-term metabolic outcomes should be assessed to facilitate their translation into precise perinatal risk stratification tools.

## Data Availability

The raw data supporting the conclusions of this article will be made available by the authors, without undue reservation.
